# Ginsenoside Rg1 Regulates the Activation of Astrocytes Through lncRNA‐Malat1/miR‐124‐3p/Lamc1 Axis Driving PI3K/AKT Signaling Pathway, Promoting the Repair of Spinal Cord Injury

**DOI:** 10.1111/cns.70103

**Published:** 2024-11-03

**Authors:** Yin Zhu, Wenjun Zou, Baihan Sun, Kelv Shen, Feiyun Xia, Hao Wang, Fengxian Jiang, Zhengfeng Lu

**Affiliations:** ^1^ Department of Orthopedics The Second Affiliated Hospital of Soochow University Suzhou China; ^2^ Department of Orthopedics The Affiliated Zhangjiagang Hospital of Soochow University Zhangjiagang China; ^3^ Department of Orthopedics Xuzhou City Hospital of TCM Xuzhou China

**Keywords:** astrocytes, ginsenoside Rg1, Lamc1, Malat1, miR‐124‐3p, PI3K/AKT, spinal cord injuries

## Abstract

**Aim:**

To investigate the regulation of ginsenoside Rg1 on the PI3K/AKT pathway through the lncRNA‐Malat1/miR‐124‐3p/ Laminin gamma1 (Lamc1) axis, activating astrocytes (As) to promote the repair of spinal cord injury (SCI).

**Methods:**

Bioinformatics analysis was used to predict miRNA targeting Lamc1 and lncRNA targeting miR‐124‐3p, which were then validated through a dual‐luciferase assay. Following transfection, the relationships between Malat1, miR‐124‐3p, and Lamc1 expression levels were assessed by qRT‐PCR and Western blot (WB). Immunofluorescence staining and immunohistochemistry were utilized to measure Lamc1 expression, while changes in cavity area were observed through hematoxylin–eosin (HE) staining. Basso‐Beattie‐Bresnahan (BBB) scale and footprint analysis were used to evaluate functional recovery. WB was performed to assess the expression of PI3K/AKT pathway‐related protein.

**Results:**

Rg1 was found to upregulate Malat1 expression, which in turn modulated the Malat1/miR‐124‐3p/Lamc1 axis. Furthermore, Rg1 activated the PI3K/Akt signaling pathway, significantly reducing the SCI cavity area and improving hind limb motor function. However, knockout of Malat1 hindered these effects, and inhibition of miR‐124‐3p reversed the silencing effects of Malat1.

**Conclusions:**

Rg1 can induce Malat1 expression to activate the Lamc1/PI3K/AKT signaling pathway by sponging with miR‐124‐3p, thereby regulating As activity to repair SCI.

## Graphical abstract

This study shows that ginsenoside Rg1 induces the activation of PI3K/AKT pathway through the lncRNA‐Malat1/miR‐124‐3p/Lamc1 axis, which is vital in controlling AS function and revealing new regulatory pathways implicated in the repair of SCI.

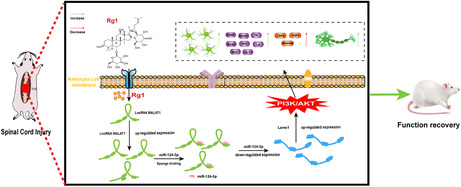

## 1 Introduction

After spinal cord injury (SCI), astrocytes (As) are activated into reactive As, subsequently forming glial scars that impede axon regeneration [1]. However, As also play a crucial role in promoting nerve repair [2]. This dual function of As could be a pivotal aspect in SCI recovery [3]. Therefore, investigating a factor or medication that can modulate As activity, counteract their negative impact, and enhance their natural repair abilities in SCI could introduce a novel theory and approach to SCI treatment.

With a rich history in traditional Chinese medicine, Ginseng contains the active component ginsenoside Rg1 [4]. Extensively studied, Rg1 has demonstrated protective properties on the cardiovascular, immune, and nervous systems [5, 6, 7]. Prior research of this study has demonstrated that Rg1 enhanced the movement, attachment, and growth of olfactory ensheathing cells through the PI3K/AKT signaling pathway, influencing various cytokines and regulating the expression of surface antigens like nestin, GLT1, and NG2. Additionally, Rg1 downregulated the expression of AQP4, CSPG, and Vimentin, while promoting the transdifferentiation of As into neuron‐like cells and anti‐apoptotic effects, all of which contributed positively to SCI repair [8, 9, 10, 11, 12, 13, 14]. Therefore, Rg1 regulates neurotrophic factors and extracellular matrix secretion in the setting of AS. This could potentially aid in the recovery of SCI by activating the PI3K/AKT pathway [15]. With this in mind, further exploration of the precise molecular mechanisms on how Rg1 exactly controls the PI3K/AKT signaling pathway need to be performed urgently.

Laminin gamma1 (Lamc1) is an essential glycoprotein for the structural integrity of the extracellular matrix involving various functions in the central nervous system (CNS) [16]. The outcome of KEGG pathway examination showed that Lamc1 functioned as a gene upstream of the PI3K/AKT signaling pathway, potentially enhancing PI3K/AKT activity through overexpression. Considering that microRNAs (miRNAs) can precisely target specific messenger RNAs (mRNAs) to affect the occurrence of various diseases, whether specific miRNAs can regulate Lamc1 needs to be explored to clarify its specific mechanism.

MiR‐124, abundantly present in the nervous system, especially in spinal cord tissue, exhibits notably elevated quantities compared to non‐nervous tissue. This supports its unique targeting of CNS tissue [17, 18]. In various cellular processes, miR‐124‐3p has been implicated including activation, proliferation, migration, differentiation, neuroprotection, and CNS regeneration [19, 20]. Research indicates that the decrease in the levels of miR‐124‐3p triggered the activation of the PI3K/AKT pathway after CNS trauma, which is essential for the healing processes associated with spinal cord damage [21, 22]. However, the potential of Rg1 enhancing Lamc1 activity, thereby modulating As activity to aid in SCI repair through targeting specific miRNAs, requires further investigation.

It is well established that long noncoding RNAs (lncRNA) can competitively bind to miRNA and regulate the miRNA/mRNA axis and downstream signal transduction [23, 24]. After experiencing a traumatic brain injury, there is a significant rise in the expression of lncRNA‐Malat1, which results in the prevention of neuronal cell death and provides neuroprotective benefits [25]. Additionally, studies indicate that altering the expression of Malat1 following sudden spinal cord trauma can effectively inhibit the inflammatory reaction of microglia, consequently slowing down the advancement of the injury [26]. Nonetheless, it remains uncertain whether the upregulation of Lamc1 by Rg1 is also influenced by the lncRNA/miRNA axis, consequently affecting the activity of As and promoting SCI repair.

Based on previous studies, this research investigated the role of the Malat1/miR‐124‐3p axis as a key focal point. Through a series of experiments, both in cell cultures and animals, this research has shown that Rg1 induces the activation of PI3K/AKT pathway through the lncRNA‐Malat1/miR‐124‐3p/Lamc1 axis, which is vital in controlling As function and revealing new regulatory pathways implicated in the repair of SCI.

## 2 Materials and Methods

### 2.1 Isolation and Culture of Primary Astrocytes as well as Rg1 Preparation

Ethical approval for the experimental procedures was obtained from the Committee of Animal Care and Ethics at Soochow University's Animal Facility in Jiangsu, China. Emphasis was placed on minimizing animal use and alleviating their distress. Sprague–Dawley (SD) rats were supplied by Zhaoyan Co. Primary As were derived from neonatal SD rats aged 1–3 days. As were then placed in six‐well plates for further experimentation, following confirmation that the proportion of GFAP‐positive cells exceeded 95% of the total cell count. The optimal concentration of Rg1 (40 μg/mL) for cell treatment was established through a CCK‐8 test, as documented in a previous investigation.

### 2.2 Cell Model Preparation

Lipopolysaccharides (LPS, MCE, and HY‐D1056) were utilized to mimic inflammation release post‐SCI and induce As injury models in vitro. As reaching 70% confluence were exposed to 1.0 μg/mL LPS for 24 h. Subsequently, the cells were utilized in the following experiments.

### 2.3 Bioinformatics Analysis

The possible interaction between miR‐124‐3p and Malat1 was predicted using Star Base (http://starbase.sysu.edu.cn/). Target Scan (https://www.targetscan.org/vert_80/) was used to anticipate the potential binding of miR‐124‐3p and Lamc1. In order to reveal potential connections among them, a luciferase reporter gene experiment was performed.

### 2.4 Dual‐Luciferase Reporter Gene Assay

The arrangements of the wild and mutant binding regions (WT‐Malat1, MUT‐Malat1, WT‐Lamc1, and MUT‐Lamc1) were planned, manufactured, and integrated into the psicheck‐2 vector for transfecting As with the miR‐124‐3p mimic (20 pmol) or mimic negative control (NC). Cells were then collected, lysed, and the luciferase activity was determined following the guidance of the dual‐luciferase reporter test (E1910; Promega), with each step being replicated three times.

### 2.5 Cell Transfection

In this investigation, the siRNA was utilized to hinder the expression of Malat1. Lipofectamine 3000 (Invitrogen, Missouri, USA) functioned as the transfection reagent for the cells. Cells were grown until they reached 70% confluence, followed by combining 1 μg of construct with 1 μL Lipofectamine 3000 in a 1:1 ratio. Post 48 h of transfection, cells were collected for further evaluation, and qRT‐PCR was performed to validate the transfection efficiency.

### 2.6 Adeno‐Associated Virus (AAV) Administration

AAV‐Malat1 (OBIO, Shanghai, China) was utilized for the knockdown of Malat1, while AAV‐enhanced green fluorescent protein (AAV‐EGFP) served as the NC (OBIO, Shanghai, China). Following anesthesia, each rat's back was flexed, and a 10 μL microinjector was inserted at the T10 spinal level of the subarachnoid space. A total of 10 μL of AAV‐Malat1 or AAV‐EGFP was gently delivered into the lateral ventricle on day 28 before SCI, and rats displaying normal motor function were selected. The initial titers of AAV‐Malat1 contained 8.73 × 10^12^ VG/mL.

MiR‐124‐3p antagomir (antagomir) (OBIO, Shanghai, China) was intrathecally injected into Malat1 knockdown rats at 24 h intervals 3 days before the SCI surgery, with antagomir‐NC as the NC (OBIO, Shanghai, China), and rats with normal motor function were molded.

### 2.7 Preparation of Rat SCI Model and Grouping

Allen's weight‐dropping technique was utilized as described in previous studies to make the SCI model [8]. Upon anesthetization with 2% halothane, laminectomy was performed to expose the spinal cord at the spinal T10 segments. Subsequently, a 10 g Kirschner wire was dropped from a height of 40 mm to initiate a weight‐drop impact. After the crash, the muscles and skin were promptly stitched together.

Sham group: Rats underwent laminectomy; SCI group: Rats with SCI daily received an i.p. injection of a vehicle solution (saline) equal to the Rg1 dose; SCI + Rg1 group: Rats with SCI received an i.p. injection of 10 mg/mL Rg1 dissolved in saline (10 mL/kg body weight); SCI + si‐Malat1 + Rg1 group: Rats were administered intrathecally with a Malat1 knockdown AAV 28 days before SCI and with Rg1 treatment; SCI + si‐NC + Rg1 group: Rats received intrathecal injection of empty vectors used as the NC 28 days before SCI and with Rg1 treatment; SCI + si‐Malat1 + Rg1 + antagomir group: Rats received intrathecal injection of Malat1 knockdown AAV and miR‐124‐3p antagomir before SCI and with Rg1 treatment; SCI + si‐Malat1 + Rg1 + NC group: Rats received intrathecal injection of Malat1 knockdown AAV and antagomir‐NC as the NC before SCI and with Rg1 treatment.

### 2.8 Functional Behavior Assessment

Motor function post‐SCI was evaluated using the Basso–Beattie–Bresnahan (BBB) Locomotor Rating Scale and footprint analysis. BBB scores were utilized to assess functional recovery preoperatively and at 1, 3, 7, 14, 21, and 28 days post‐injury (dpi). An observer assigned a score within a range from 0 to 21. Footprint analysis entailed examining motor coordination by submerging the hindlimb in blue dye and the forelimb in red dye. Rats were permitted to ambulate along a straight strip of white paper, after which their footprints underwent analysis.

### 2.9 Quantitative Reverse Transcription Polymerase Chain Reaction (qRT‐PCR) Analysis

LncRNAs and gene expressions were normalized to GAPDH, while miRNA levels were referenced to U6. Details of the primer sequences are provided in Table 1.

**TABLE 1:** qRT‐PCR results.

Target gene	Forward primers, 5′–3′	Reverse primers, 5′–3′
lncR‐Malat1	CTATGCTGTTGGCACGACA	TCCTGAGGTGACTGTGAACC
miR‐124‐3p	CGCGTGTTCACAGCGGAC	AGTGCAGGGTCCGAGGTATT
Lamc1	CTTGCGACTGTCACCACGAAGG	TCACACTGGTCACAGCGATTGC
U6	GCTTCGGCAGCACATATACTAAAAT	CGCTTCACGAATTTGCGTGTCAT
GAPDH	GACATGCCGCCTGGAGAAAC	AGCCCAGGATGCCCTTTAGT

### 2.10 Western Blot (WB) Analysis

The As and fresh spinal cord tissue samples were obtained, and the total protein was extracted for Western blot (Data S1).

### 2.11 Immunofluorescence Staining

The As and spinal cord sections were obtained and subjected to immunofluorescence staining (Data S1).

### 2.12 Immunohistochemical Analysis

Spinal cord sections were obtained and subjected to immunohistochemical analysis (Data S1).

### 2.13 Hematoxylin–Eosin (HE) Staining

Spinal cord sections were similarly obtained and subjected to hematoxylin–eosin staining (Data S1).

### 2.14 Statistical Analysis

Mean ± SD values from three samples were used for all measured data and then analyzed using GraphPad Prism 8 software. The normality of the data was checked with the Shapiro–Wilk test. Statistical significance of differences between two groups was determined by Student's *t* test, while that among multiple groups was determined by the one‐way or two‐way analysis of variance (ANOVA). For various analyses, *p* < 0.05 was considered statistically significant.

## 3 Result

### 3.1 Malat1 and Lamc1 Were Elevated in SCI Tissues and Injured As, While miR‐124‐3p Was Reduced

**FIGURE 1:**
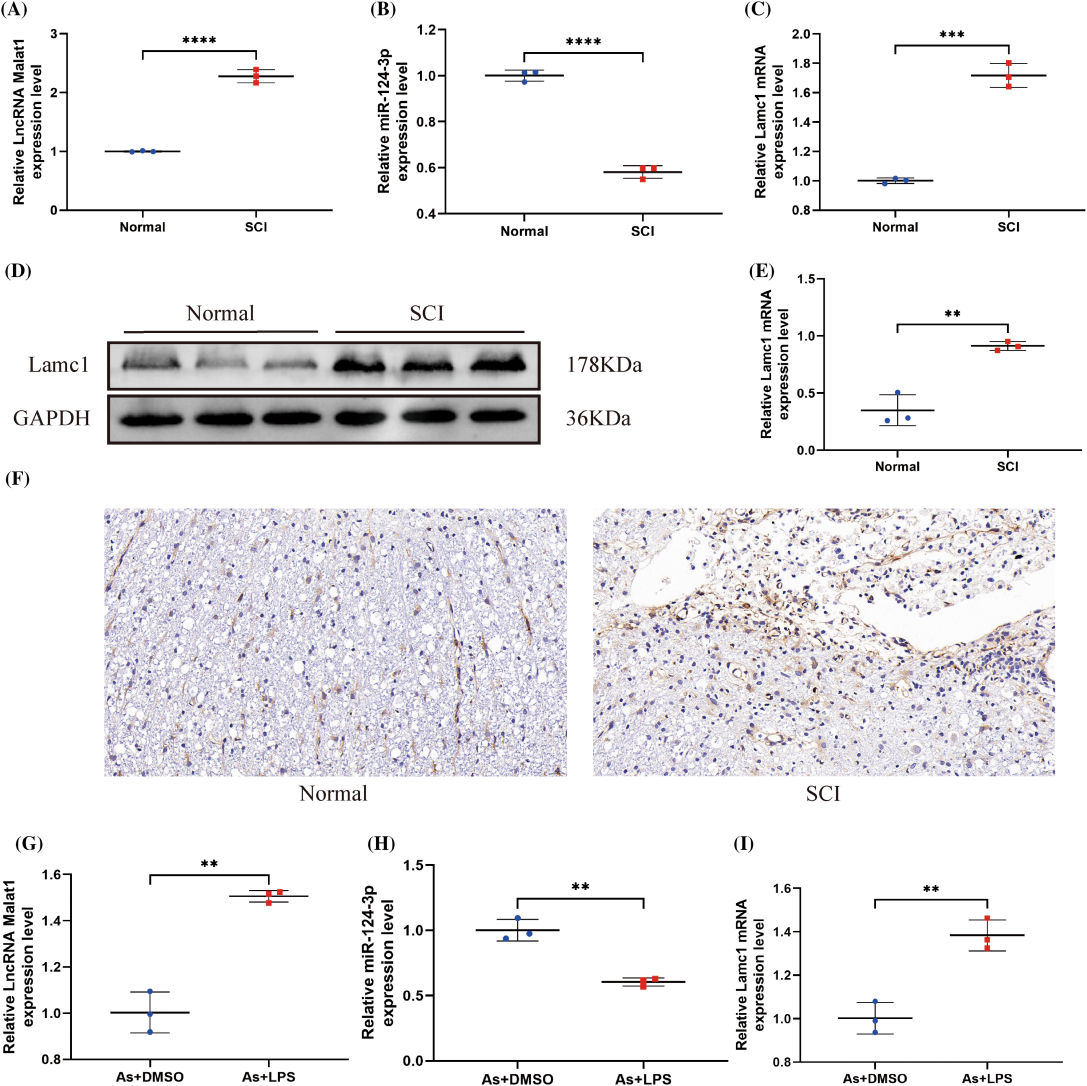
Malat1 and Lamc1 were highly expressed and were negatively correlated to miR‐124‐3p, which was lowly expressed post‐injury. (A–C) Analysis using qRT‐PCR was performed to examine the expression levels of Malat1, miR‐124‐3p, and Lamc1 in spinal cord tissues. (A) ****p < 0.0001, (B) ****p < 0.0001, and (C) ***p = 0.0001 < 0.001 versus normal. (D–E) Detection of Lamc1 expression in spinal cord tissues using WB, followed by densitometric quantitation. (E) **p = 0.0023 < 0.01 versus normal. (F) Evaluation of Lamc1 levels in spinal cord tissues was conducted through immunohistochemistry staining analysis. (G–I) Expression levels of Malat1, miR‐124‐3p, and Lamc1 in normal and injured As were determined using qRT‐PCR analysis. (**p < 0.01, ***p < 0.001, and ****p < 0.0001. Significance was determined by one‐way ANOVA, followed by Tukey's multiple comparison test. The sample size was *n* = 3). (G) ***p = 0.0007 < 0.001, (H) **p = 0.0014 < 0.01, and (I) **p = 0.0029 < 0.01 versus As+DMSO (*p* values were calculated with Student's *t* test, *n* = 3).

In order to investigate the roles of Malat1, miR‐124‐3p, and Lamc1 in SCI, the expression levels were initially evaluated in both SCI tissue and cells. Elevated Malat1 levels were observed in rat SCI tissues, whereas miR‐124‐3p levels were decreased compared to normal spinal cord tissues (Figure 1A,B). Similarly, Lamc1 mRNA and protein levels increased in SCI tissues (Figure 1C–E). Additionally, comparing LPS‐treated As with normal As revealed a similar pattern. In LPS‐treated As, Malat1 and Lamc1 mRNA levels were significantly higher, while miR‐124‐3p expression was markedly reduced. Consequently, As were selected for further experiments. These results suggest an upregulation of Malat1 and Lamc1, as well as a downregulation of miR‐124‐3p, in both SCI and injured As.

### 3.2 Malat1 Directly Bound miR‐124‐3p, While miR‐124‐3p Targeted Lamc1

**FIGURE 2:**
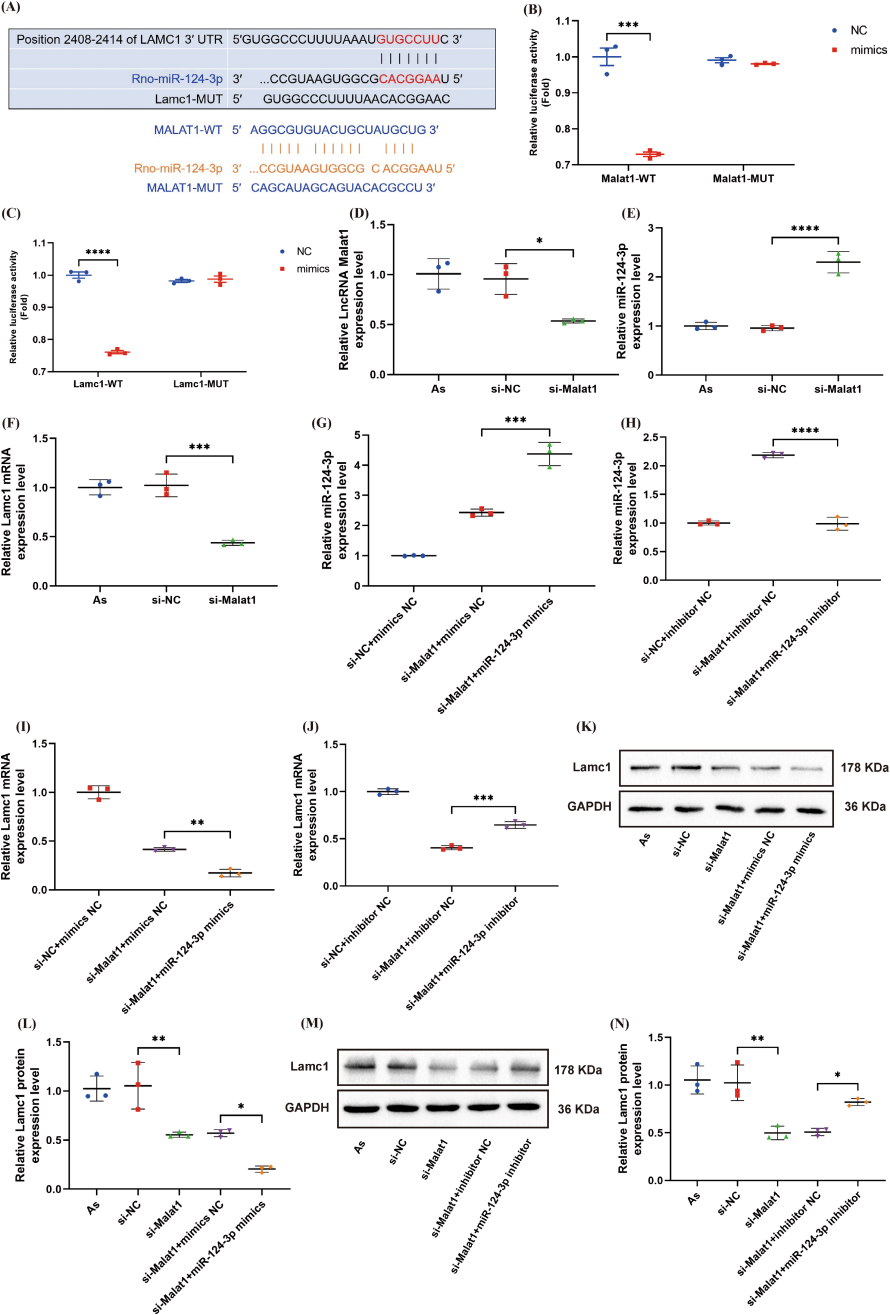
Malat1 targeted miR‐124‐3p and regulated the expression of Lamc1 by ceRNA mechanism. (A) Star Base predicted the putative binding site of miR‐124‐3p with Malat1, while Target Scan identified potential miR‐124‐3p binding sites within Lamc1‐3′‐UTR. (B, C) In HEK293T cells, the dual‐luciferase reporter assay confirmed the interaction between Malat1 and miR‐124‐3p, as well as the targeting of Lamc1 by miR‐124‐3p. ***p = 0.0004 < 0.001 and ****p < 0.0001versus NC. (D–J) Following transfection, qRT‐PCR analysis of As was performed to assess the expression levels of Malat1, miR‐124‐3p, and Lamc1, using GAPDH or U6 as internal controls. (D–F) *p = 0.0149 < 0.05, ****p < 0.0001, and ***p = 0.0003 < 0.001 versus si‐NC. (G–J) ***p = 0.0001 < 0.001, ****p < 0.0001, **p = 0.0016 < 0.01, and ***p = 0.0002 < 0.001 versus si‐Malat1 + mimics NC. (K–N) Expression of Lamc1 in As post‐transfection was evaluated through WB and densitometric quantitation, with GAPDH serving as an internal control. (L) **p = 0.0039 < 0.01 versus si‐NC and *p = 0.0288 < 0.05 versus si‐Malat1 + mimics NC. (N) **p = 0.0015 < 0.01 versus si‐NC and *p = 0.0442 < 0.05 versus si‐Malat1 + mimics NC. (*p* values of (B, C) were calculated with Student's *t* test, and the other *p* values were calculated with one‐way ANOVA, followed by Tukey's multiple comparisons test; *n* = 3).

LncRNAs act as sponges for miRNAs, exerting their effects by targeting downstream mRNAs. This study aimed to explore the regulatory interactions among Malat1, miR‐124‐3p, and Lamc1. By altering the levels of Malat1 and miR‐124‐3p, the influence on Lamc1 expression was investigated. According to bioinformatics analysis, miR‐124‐3p is predicted to be a target of Malat1, and Lamc1 is a target gene of miR‐124‐3p (Figure 2A). Luciferase assays conducted in HEK293T cells demonstrated that transfection with miR‐124‐3p mimic and WT‐Malat1 or WT‐Lamc1 led to reduced luciferase activity compared to the control group. Conversely, transfection with MUT‐Malat1 or MUT‐Lamc1 did not show a significant difference (Figure 2B,C). These results suggest that Malat1 and Lamc1 can interact with miR‐124‐3p directly.

The transfection of As with si‐Malat1 resulted in a significant reduction in Malat1 levels, as demonstrated by qRT‐PCR (Figure 2D). Furthermore, there was a noticeable increase in miR‐124‐3p levels, accompanied by a decrease in Lamc1 mRNA in cells transfected with si‐Malat1 compared to those transfected with si‐NC (Figure 2E,F). Subsequent transfections of miR‐124‐3p mimics or inhibitors into Malat1‐knockdown As further confirmed the regulatory role of Malat1 on Lamc1. The results illustrated that transfection of miR‐124‐3p mimics led to an upregulation of miR‐124‐3p expression, whereas transfection of inhibitors resulted in the downregulation of miR‐124‐3p, indicating the success of the transfections (Figure 2G,H). Additionally, miR‐124‐3p mimics were capable of further reducing the already low expression level of Lamc1 post‐Malat1 knockdown, while miR‐124‐3p inhibitors partially restored Lamc1 expression following Malat1 knockdown, as evidenced by qRT‐PCR (Figure 2I,J). By these observations, WB showed a reduction in Lamc1 expression after si‐Malat1 transfection, with a further decrease observed after co‐transfection with miR‐124‐3p mimics and an increase in Lamc1 expression after co‐transfection with miR‐124‐3p inhibitors (Figure 2K–N). These findings indicate that Malat1 negatively influences miR‐124‐3p, which consequently positively impacts Lamc1 in As, revealing a potential mechanism by which Malat1 modulates Lamc1 expression through competitive binding with miR‐124‐3p.

### 3.3 Rg1 Targeted Malat1 and Regulated the Malat1/miR‐124‐3p/Lamc1 Axis in Injured As

**FIGURE 3:**
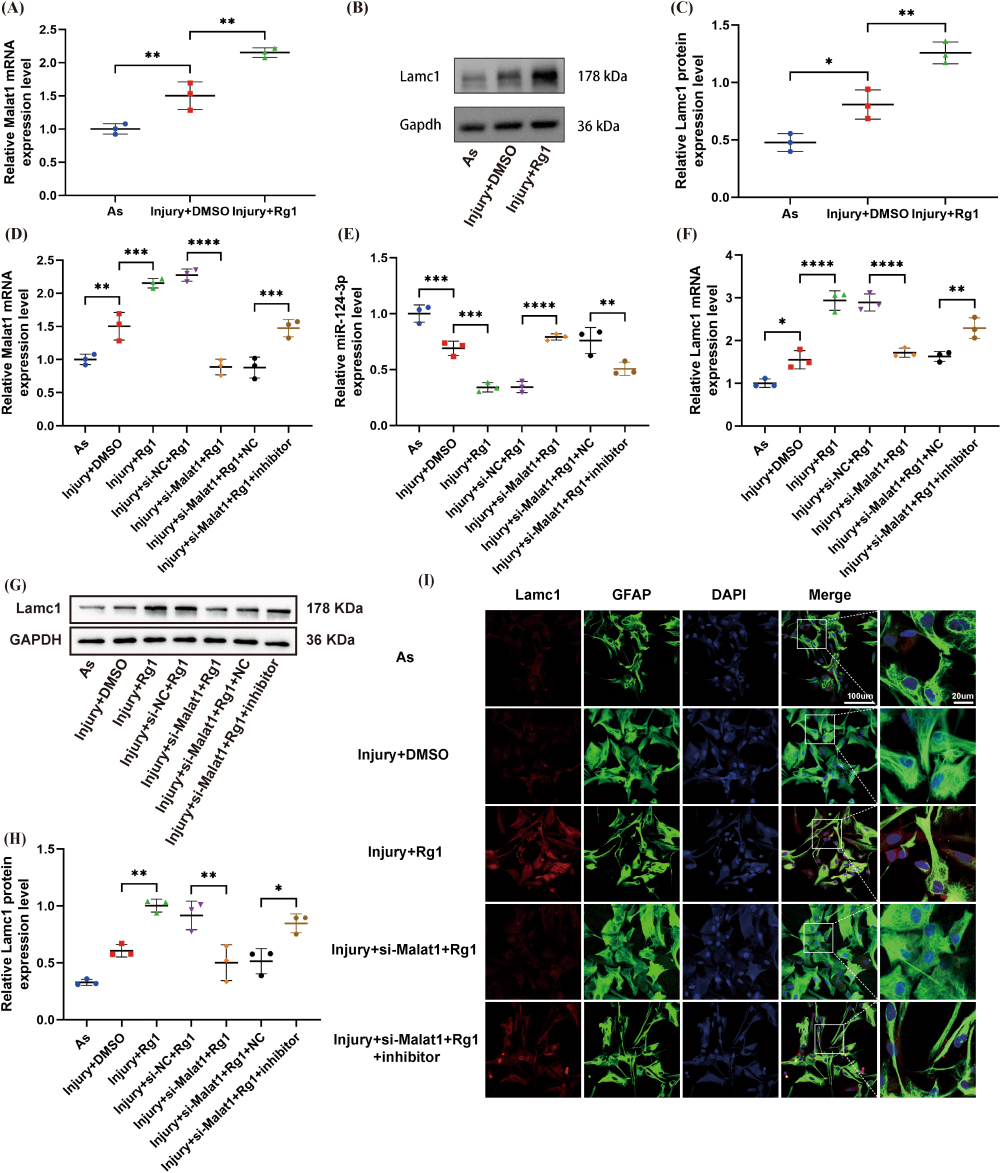
Rg1 targeted Malat1 regulated the Malat1/miR‐124‐3p/Lamc1 axis by ginsenoside Rg1 in vitro. (A) Evaluating Malat1 expression in As post‐LPS treatment with or without incubation with DMSO or Rg1 using qRT‐PCR analysis, with GAPDH as the internal control. **p = 0.0092 < 0.01 versus As and **p = 0.0025 < 0.01 versus Injury+DMSO. (B, C) Employing WB and quantitative analysis to assess Lamc1 expression in As post‐LPS treatment with or without exposure to DMSO or Rg1, utilizing GAPDH as the internal control. (C) *p = 0.0177 < 0.05 versus As and **p = 0.0041 < 0.01 versus Injury+DMSO. (D–F) Conducting qRT‐PCR analysis to examine the levels of Malat1, miR‐124‐3p, and Lamc1 in As post‐transfection, post‐LPS treatment, and post‐incubation with DMSO or Rg1, using either GAPDH or U6 as internal controls. (D) **p = 0.0047 < 0.01 versus RAs, ***p = 0.0004 < 0.001 versus Injury+DMSO, ****p < 0.0001 versus Injury+si‐NC + Rg1, and ***p = 0.0010 < 0.001 versus Injury+si‐NC + Rg1 + NC. (E) ***p = 0.0010 < 0.001 versus RAs, ***p = 0.0003 < 0.001 versus Injury+DMSO, ****p < 0.0001 versus Injury+si‐NC + Rg1, and **p = 0.0063 < 0.001 versus Injury+si‐NC + Rg1 + NC. (F) *p = 0.0297 < 0.01 versus RAs, ****p < 0.0001 versus Injury+DMSO, ****p < 0.0001 versus Injury+si‐NC + Rg1, and **p = 0.0076 < 0.01 versus Injury+si‐NC + Rg1 + NC. (G, H) Utilizing WB and quantitative analysis to investigate Lamc1 expression in As post‐transfection, post‐LPS treatment, and post‐incubation with DMSO or Rg1, with GAPDH as the internal control. (H) **p = 0.0029 < 0.01 versus Injury+DMSO, **p = 0.0019 < 0.01 versus Injury+si‐NC + Rg1, and *p = 0.0124 < 0.05 versus Injury+si‐Malat1 + Rg1 + NC. (I) Performing immunofluorescence staining to evaluate Lamc1 expression in As post‐transfection, post‐LPS treatment, and post‐incubation with DMSO or Rg1, with scale bars provided for reference (left: 100 μm; right: 20 μm) (*p* values were calculated with one‐way ANOVA, followed by Tukey's multiple comparisons test; *n* = 3).

Given that the potential of Rg1 to regulate lncRNAs in SCI remains to be established. As previously mentioned, there was a significant increase in the level of Malat1 in injured As, and notably, Rg1 further enhanced the expression of Malat1 (Figure 3A). Similarly, the results from WB demonstrated that Rg1 effectively elevated the expression of Lamc1 in injured As as well (Figure 3B,C). Consequently, Malat1 became the focus of our further studies on subsequent mechanistics. In order to elucidate the role of Rg1 in injured As, miR‐124‐3p inhibitor was introduced into Malat1‐knockdown cells to investigate its influence on the Malat1/miR‐124‐3p/Lamc1 pathway in injured As.

The qRT‐PCR findings revealed that Rg1 could further enhance the expression of Malat1. However, when Malat1‐knocked down As were treated with Rg1, there was no increase in Malat1 expression. Notably, the reduced expression of Malat1 was partially restored following transfection with the miR‐124‐3p inhibitor (Figure 3D–F). Furthermore, WB and immunofluorescence staining results indicated that Rg1 could augment the protein expression and fluorescence intensity of Lamc1. After Malat1 knockdown, however, Rg1 could not enhance the protein expression and fluorescence intensity of Lamc1. Nevertheless, transfection with the miR‐124‐3p inhibitor partially reversed the diminished levels of Lamc1(Figure 3G–I).

In conclusion, these findings highlighted that Malat1 served as a direct target of Rg1 and acted as a critical player in the Malat1/miR‐124‐3p/Lamc1 axis. Rg1 demonstrated the ability to elevate the expression of Malat1 and Lamc1, a response attenuated by si‐Malat1 transfection. Additionally, co‐transfection with the miR‐124‐3p inhibitor counteracted the impacts of Malat1 knockdown in vitro.

### 3.4 Rg1 Regulates the Malat1/miR‐124‐3p/Lamc1 Axis in the Rat SCI Model

**FIGURE 4:**
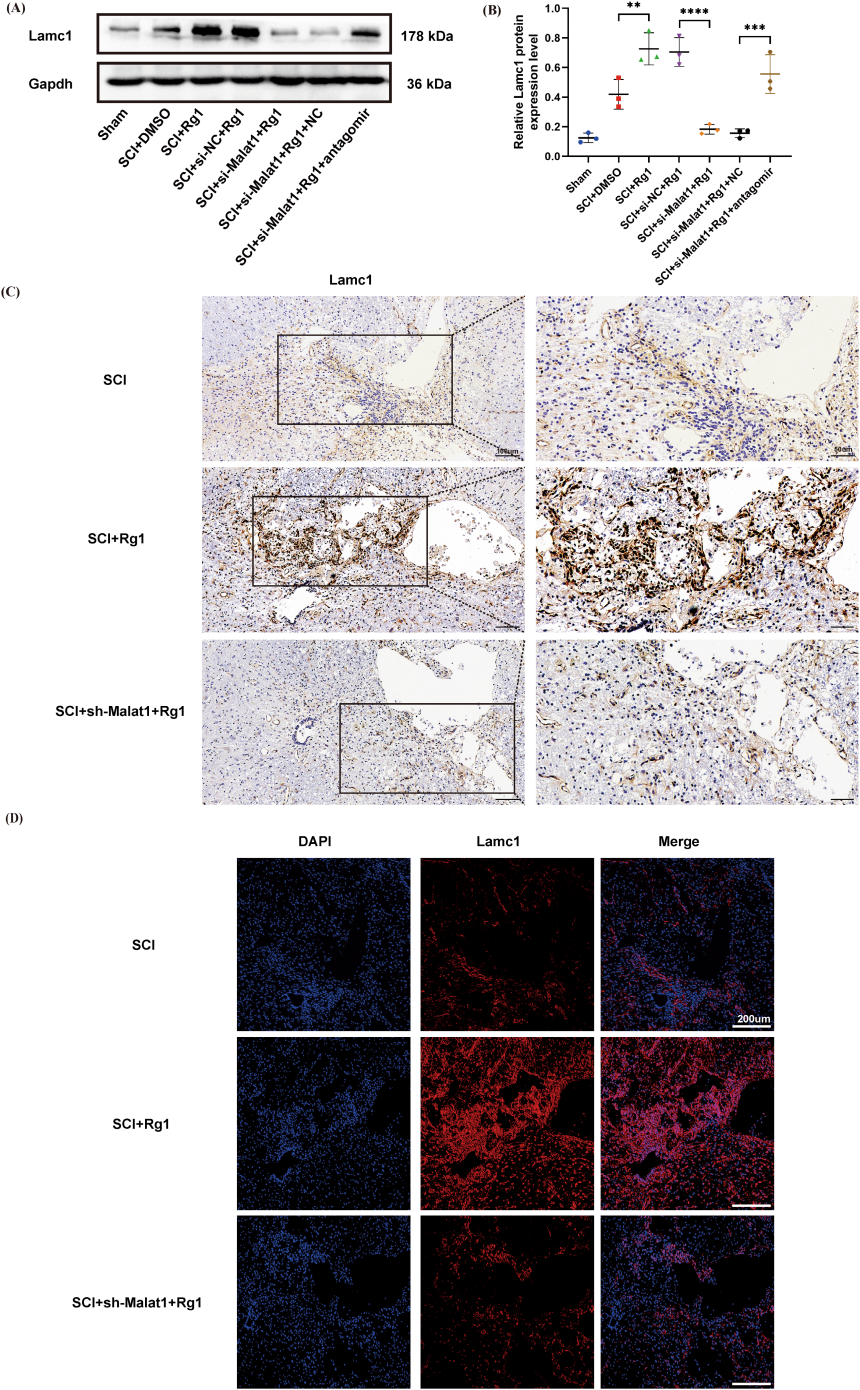
Regulation of the Malat1/miR‐124‐3p/Lamc1 axis by ginsenoside Rg1 in vivo. (A, B) Different groups were subjected to WB and quantitative analysis to assess Lamc1 expression. (B) **p = 0.0086 < 0.01 versus SCI + DMSO, ****p < 0.0001 versus SCI + si‐NC + Rg1, and ***p = 0.0008 < 0.001 versus SCI + si‐Malat1 + Rg1 + NC. (C) Images representing immunohistochemical staining analysis of Lamc1 in the SCI group, the SCI + Rg1 treatment group, and the SCI + si‐Malat1 + Rg1 treatment group (left scale bar = 100 μm; right scale bar = 50 μm). (D) Immunofluorescence staining analysis of Lamc1 in the SCI group, the SCI + Rg1 treatment group, and SCI + si‐Malat1 + Rg1 treatment group displayed in the representative images (scale bar = 200 μm). (*p* values were calculated with one‐way ANOVA, followed by Tukey's multiple comparisons test; *n* = 3).

Subsequent trials illustrated the regulating function of Rg1 within the Malat1/miR‐124‐3p/Lamc1 pathway in SCI rats 14 dpi. Examination through WB demonstrated an augmentation in Lamc1 protein levels in the SCI + Rg1‐treated group compared to the SCI group. Meanwhile, a noticeable decrease in Lamc1 expression was observed in the SCI + si‐Malat1 + Rg1‐treated group compared to the SCI + si‐NC + Rg1‐treated group. Additionally, Lamc1 levels showed a significant increase in the SCI + si‐Malat1 + Rg1‐treated+miR‐124‐3p‐antagomir group as opposed to the SCI + si‐Malat1 + Rg1‐treated+antagomir NC group (Figure 4A,B). The results from immunohistochemistry further confirmed these discoveries, illustrating an elevation of Lamc1 in the SCI + Rg1‐treated group and a suppression in the SCI + si‐Malat1 + Rg1‐treated group in vivo (Figure 4C). Corresponding outcomes were replicated in immunofluorescence staining (Figure 4D). In conclusion, the data implied that Rg1 promoted an increase in Lamc1 expression. Nevertheless, this impact was hindered by si‐Malat1 transfection but can be reversed by co‐transfection with miR‐124‐3p antagomir in vivo.

### 3.5 Rg1 Treatment Relieves Histological and Functional Disorders via Malat1/miR‐124‐3p/Lamc1 Axis In Vivo

**FIGURE 5:**
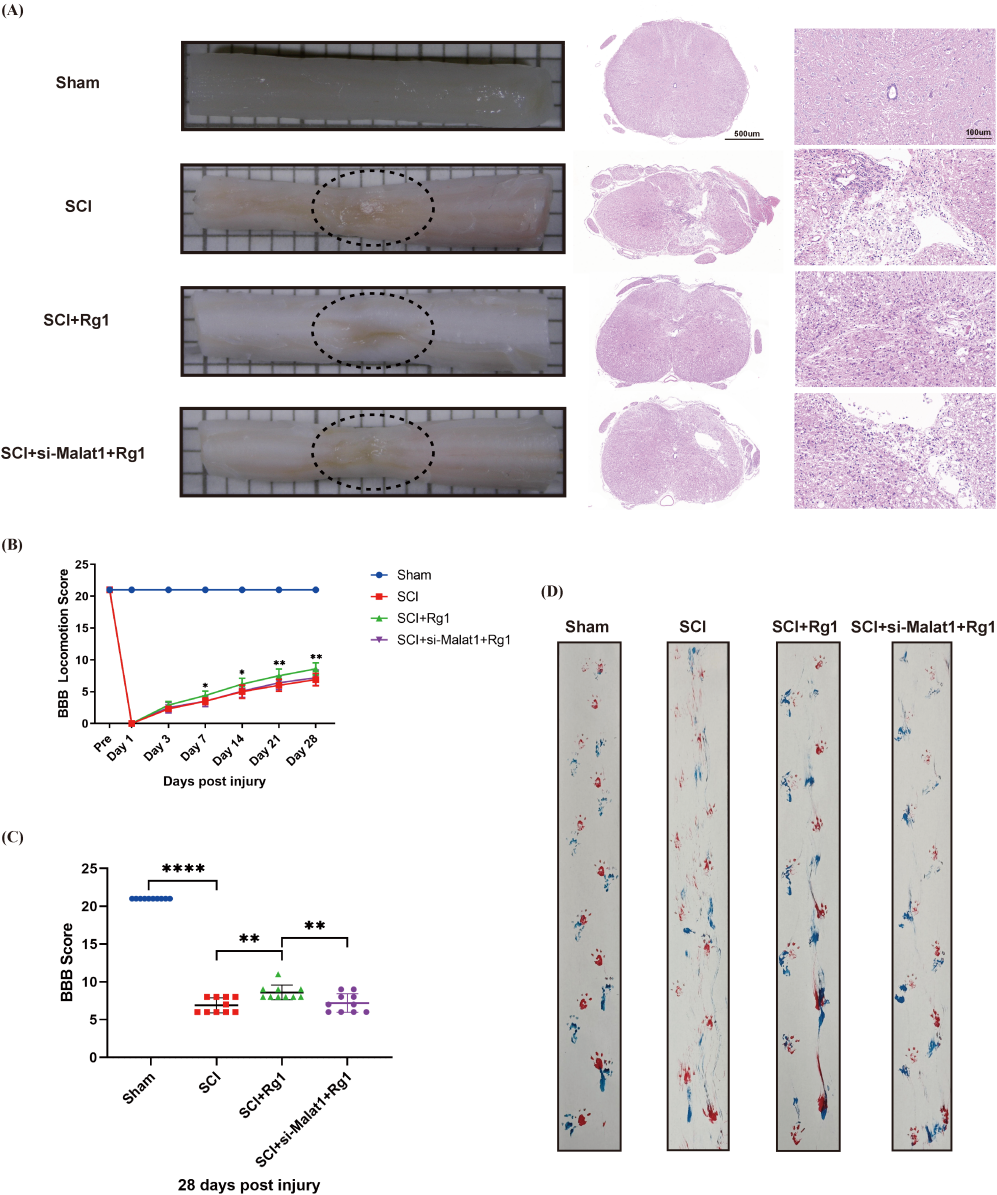
Promotion of histological and functional recovery through the Malat1/miR‐124‐3p/Lamc1 axis by ginsenoside Rg1. (A) HE staining and representative images of the spinal cord were conducted on cross sections of the injury epicenter 28 dpi. The blue nuclei and red cytoplasm are visible, with black circles indicating the area of SCI. Scale bars on the middle and right sides represent 500 μm and 100 μm, respectively. (B) Motor function evaluation post‐SCI was done using the BBB score in the Sham, SCI, SCI + Rg1, and SCI + si‐Malat1 + Rg1 groups at specified time points. The quantification of BBB exercise rating scale scores on day 28 was performed for each group. *p = 0.0113 < 0.05 versus SCI (7 dpi), *p = 0.0175 < 0.05 versus SCI (14 dpi), **p = 0.0034 < 0.01 versus SCI (21 dpi), and **p = 0.0091 < 0.01 versus SCI (28 dpi). (C) Quantification of BBB exercise rating scale scores on day 28 was performed for each group. ****p < 0.0001 versus Sham, **p = 0.0012 < 0.01versus SCI, and **p = 0.0091 < 0.01 versus SCI + si‐Malat1+ Rg1. (D) Findings of the footprint analysis experiments conducted on the various groups are presented. (*p* values of (B) were analyzed by two‐way analysis of variance, and *p* values of (C) were calculated with one‐way ANOVA, followed by Tukey's multiple comparisons test; *n* = 10).

In order to explore the potential of Rg1 in decreasing lesion cavity size and enhancing recovery of movement via Malat1‐related pathways, an examination using HE staining was performed on transverse slices of the injury center. Rats were then assessed with BBB score and gait analysis on day 28 following SCI. Furthermore, a Malat1 adeno‐associated virus was applied to decrease the expression of Malat1, aiming to investigate its function in mediating the inhibitory impacts of Rg1 on SCI. Gross images and HE staining were employed to detect variations in the SCI region 28 dpi. The spinal cord tissues in the Sham group exhibited typical features, while the SCI group demonstrated structural disarray and cavity creation. The SCI + Rg1 treatment group displayed reduced cavities. Conversely, the SCI + si‐Malat1 + Rg1 treatment group exhibited an increase in vesicle‐like structures compared to the SCI + Rg1 treatment group (Figure 5A). Initially, following SCI in rats, hindlimb function was lost but later regained over time. Motor function was significantly affected in all experimental groups at 1 and 3 dpi. However, by day 7, the group receiving SCI + Rg1 displayed notably improved motor recovery compared to the group with only SCI, whereas there was no significant contrast between the SCI + si‐Malat1 + Rg1 group and the SCI group (Figure 5B). On the 28th day, a noticeable gap in motor function recovery among the groups was observed (Figure 5C). Footprint analysis revealed coordinated hindlimb movement in the Rg1 treatment group, resembling the Sham group. In contrast, the SCI group and SCI + si‐Malat1 + Rg1 group showed hindlimb dragging (Figure 5D).

These findings suggested that Rg1 promoted spinal cord tissue repair by regulating the Malat1/miR‐124‐3p/Lamc1 axis, enhancing the hind limb motor function.

### 3.6 Rg1 Activates the PI3K/AKT Signaling Pathway via the Malat1/miR‐124‐3p/Lamc1 Axis, Both In Vitro and In Vivo

Previous research has shown that Rg1‐induced activation of As enhances recovery through the PI3K/AKT signaling pathway following SCI.

To explore the involvement of the Malat1/miR‐24‐3p/Lamc1 axis in mediating Rg1's activation of the PI3K/AKT pathway, Malat1 inhibition experiments were carried out by using siRNA in laboratory and animal models.

**FIGURE 6:**
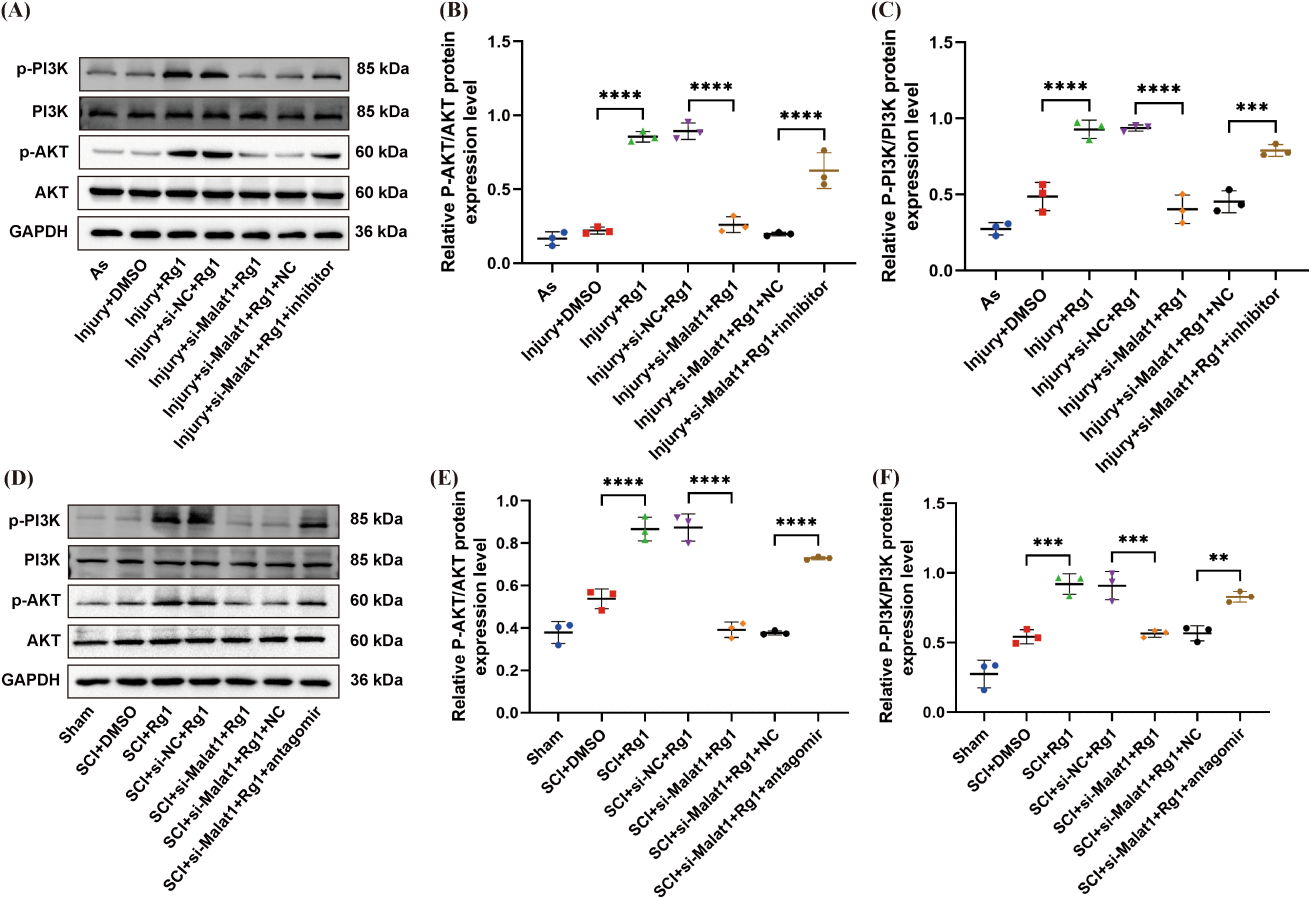
Expression profiles of components of the PI3K/AKT signaling pathway through the Malat1/miR‐124‐3p/Lamc1 axis by ginsenoside Rg1. (A–C) In vitro, WB and densitometric quantitation were conducted to evaluate the levels of proteins related to the PI3K/AKT pathway in different experimental groups. (B) ****p < 0.0001 versus Injury+DMSO, ****p < 0.0001 versus Injury+si‐Malat1 + Rg1, and ****p < 0.0001 versus Injury+si‐Malat1 + Rg1 + NC. (C) ****p < 0.0001 versus Injury+DMSO, ****p < 0.0001 versus Injury+si‐Malat1 + Rg1, and ***p = 0.0003 < 0.001 versus Injury+si‐Malat1 + Rg1 + NC. (D–F) For the in vivoβ experiments, WB and densitometric quantitation were carried out to measure the expression of proteins associated with the PI3K/AKT pathway in the diverse groups. (E) ****p < 0.0001 versus SCI + DMSO, ****p < 0.0001 versus SCI + si‐NC + Rg1, and ****p < 0.0001 versus SCI + si‐Malat1 + Rg1 + NC. (F) ***p = 0.0002 < 0.001 versus SCI + DMSO, ***p = 0.0004 < 0.001 versus SCI + si‐NC + Rg1, and **p = 0.0052 < 0.01 versus SCI + si‐Malat1 + Rg1 + NC (*p* values were calculated with one‐way ANOVA, followed by Tukey's multiple comparisons test; *n* = 3).

WB was utilized to assess levels of different PI3K/Akt pathway markers. Our data demonstrated that Rg1 treatment notably boosted phosphorylated PI3K and AKT expression, reversed with Malat1 silencing. Furthermore, transfection with a miR‐124‐3p blocker in damaged As significantly increased phosphorylated PI3K and Akt protein levels. Corresponding outcomes were observed in animal trials, where Rg1 treatment elevated phosphorylated PI3K and p‐AKT protein levels post‐SCI. (Figure 6A–C) Conversely, Malat1 suppression reduced these protein levels, while the inhibition of miR‐124‐3p expression restored phosphorylated PI3K and p‐AKT protein levels. (Figure 6D–F) Our results indicate that Rg1 triggers the PI3K/AKT pathway through the Malat1/miR‐124‐3p/Lamc1 axis in both lab settings and animal models.

## 4 Discussion

Extensively investigated in the field of cell regeneration and repair are the pathways of PI3K/Akt signaling [27]. This pathway plays crucial roles in cell metabolism, proliferation, survival, growth, cancer development, angiogenesis regulation, and disease progression [28, 29, 30, 31]. Lamc1 is an upstream gene in the PI3K/AKT signaling pathway. Previous research has demonstrated that Rg1 can upregulate Laminin (LN) expression, activate the PI3K/Akt pathway, regulate the expression of pertinent proteins in damaged As cells in a laboratory setting, improve As cell function, promote healing of scratch injuries, and enhance recovery from SCI. This study highlights the positive regulatory impact of Rg1 on the PI3K/AKT signaling pathway in promoting cell repair mechanisms. Lamc1, a subtype of LN, has been thoroughly examined in its connection to Schwann cells [32]. Research has shown that deleting the Lamc1 gene in Schwann cells through the Cre–loxP system hinders axonal regeneration, resulting in motor deficits like hind limb paralysis and tremors [33]. This highlights the vital role of Lamc1 as a matrix component in the peripheral nervous system, crucial for Schwann cell differentiation, axonal myelination, and peripheral nerve regeneration [34, 35]. Notably, the involvement of Lamc1 in SCI has not been explored. Our recent research suggests that Rg1 may increase Lamc1 levels in cases of SCI, providing insights into Lamc1's role in the PI3K/Akt signaling pathway. Furthermore, our study proposes that Rg1 could impact As activity during SCI recovery by enhancing Lamc1 expression.

miRNA is a crucial upstream regulatory gene of mRNA; several miRNA variants are considered potential biomarkers of SCI [36, 37, 38, 39]. Bioinformatics analysis has identified Lamc1 as the specific gene targeted by miR‐124‐3p, which is known to participate in competitive endogenous RNA (ceRNA) pathways in various neurological conditions. For example, miR‐124‐3p interacts with lncR HOXA11‐As to inhibit the migration and invasion of glioma cells [40]. Further research is necessary to explore the impact of miR‐124‐3p on SCI outcomes, focusing on enhancing As functionality to improve recovery. Experimental results indicate that miR‐124‐3p modulates As function by targeting Lamc1, as evidenced by luciferase activity assays and qRT‐PCR. Investigating the upstream lncRNA of miR‐124‐3p could provide valuable insights into the miR‐124‐3p/Lamc1 axis in SCI rehabilitation.

LncRNA is a type of RNA that, similar to miRNA, competes with miRNA for binding to regulate downstream signal transduction [23, 41]. Our research identified miR‐124‐3p as a binding partner for Malat1. Previous studies have linked the expression of Malat1 to neuronal activity and damage, highlighting its crucial role in the nervous system [42, 43, 44]. In line with prior studies, Malat1 has been shown to boost the migration and invasion abilities in hepatocellular carcinoma through its interaction with miR‐124‐3p [45]. Malat1 interacts with miR‐124‐3p.1/KLF5 to enhance the remodeling of pulmonary vasculature and facilitate the progression of cell cycle in pulmonary artery hypertension [46]. Nevertheless, the effects of Malat1 on AS function after SCI have not been investigated in existing research. This investigation demonstrated a notable increase in the levels of Malat1, a decrease in miR‐124‐3p expression, and an increase in Lamc1 expression within the central nervous system following SCI. Comparable results were noted in damaged AS. In vitro studies confirmed that Malat1 modulated Lamc1 expression in AS by competitively binding miR‐124‐3p.

In order to investigate the mechanism of autologous repair initiation by which Rg1 upregulated Malat1 expression post‐SCI, competed with miR‐124‐3p, indirectly upregulated Lamc1 expression, activated the PI3K/AKT pathway, and regulated AS, a series of in vitro and in vivo experiments were performed. The findings, validated by a cell injury model and a rat SCI model, from qRT‐PCR, WB, immunofluorescence staining, and immunohistochemistry staining collectively indicated that Rg1 has the ability to increase the expression of Malat1, lower the levels of miR‐124‐3p, and enhance the expression of Lamc1. Conversely, knocking out Malat1 led to an upregulation of miR‐124‐3p and a downregulation of Lamc1. In vitro and in vivo experiments have demonstrated that Rg1 activated the PI3K/AKT signaling pathway through the Malat1/miR‐124‐3p/Lamc1 axis. The transfection of miR‐124‐3p inhibitor induces the reversal of Lamc1 expression. Rg1 has been found to reduce the syrinx area in rats and enhance the hindlimb motor function recovery. Nonetheless, the inhibition of Malat1 impeded the impacts, and the downregulation of miR‐124‐3p restored the consequences of Malat1 suppression. The hindrance of axonal regeneration post‐SCI is believed to be primarily due to the inhibitory microenvironment consisting of inflammatory responses and scar tissue [47]. Regrettably, this study did not include relevant investigations to support these conclusions. In future studies, it is possible to investigate how the Malat1/miR‐124‐3p/Lamc1 pathway affects the modulation of local fibrosis within SCI sites and facilitates the regeneration of nerve axons.

The results of this study show that Rg1 promoted the PI3K/AKT signaling pathway through the Malat1/miR‐124‐3p/Lamc1 axis to modulate As function and improve SCI recovery. The comprehension of SCI mechanisms was enhanced by this study, and a robust foundation for forthcoming research was esablished.

## Author Contributions

Y.Z., W.Z., and Z.L. devised and conducted the experiments. Z.L. oversaw the research. Y.Z., W.Z., and Z.L. analyzed and interpreted data. Y.Z., W.Z., B.S., K.S., F.X., and H.W. prepared manuscript drafts. Y.Z., W.Z., B.S., K.S., F.X., H.W., F.J., and Z.L. critically revised the manuscript for significant intellectual content. All authors reviewed and endorsed the final manuscript.

## Conflicts of Interest

The authors declare no conflicts of interest.

## Supporting information


Data S1.

## Data Availability

The data that supports the findings of this study are available in the Supporting Information of this article.
