# Supplementary material for: Ginsenoside Rg1 Regulates the Activation of Astrocytes Through lncRNA‐Malat1/miR‐124‐3p/Lamc1 Axis Driving PI3K/AKT Signaling Pathway, Promoting the Repair of Spinal Cord Injury
Source: CNS Neurosci Ther. 2024 Nov 3;30(11):e70103. doi: 10.1111/cns.70103 (PMC11532020; doi:10.1111/cns.70103)
Supplement: Supplementary file 1 — Data S1. [file CNS-30-e70103-s001.docx]

**Ginsenoside Rg1 regulates the activation of astrocytes through lncRNA-Malat1/miR-124-3p/Lamc1 axis driving PI3K/AKT signaling pathway, promoting the repair of spinal cord injury**

Yin Zhu^1,2^
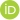
 **|** Wenjun Zou^1^ **|** Baihan Sun ^1,3^ **|** Kelv Shen ^1^ **|** Feiyun Xia ^1^ **|** Hao Wang^1^ **|** Fengxian Jiang^1^ **|** Zhengfeng Lu^1^
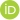


^1^ Department of orthopedics, The Second Affiliated Hospital of Soochow University, Suzhou, China

^2^ Department of orthopedics, The Affiliated Zhangjiagang Hospital of Soochow University, Zhangjiagang, China

^3^ Department of Orthopedics, Xuzhou City Hospital Of TCM, Xuzhou, China

**Corresponding author:**

Zhengfeng Lu; Department of orthopedics, The Second Affiliated Hospital of Soochow University, No. 1055 Sanxiang Road, Suzhou 215004, China.

Email: [lzf@suda.edu.cn](mailto:lzf@suda.edu.cn)

**Funding information**

This work was supported by grants from National Natural Science Foundation of China (No. 82074173); and Suzhou Science and Technology Development Project (SKYD2023052).

Yin Zhu, Wenjun Zou and Baihan Sun contributed equally to this work.

**Supplementary materials**

**Methods:**

**Western blot (WB) analysis**

The membranes were blocked with 5% BSA in tris-buffered saline with tween 20 (TBST) at room temperature for 1 hour before being incubated overnight at 4°C with primary antibodies against Anti-Laminin gamma1 (Lamc1, 1:1000, Abcam, ab233389); PI3K (1:1000, CST, 4249); p-PI3K (1:1000, CST, 4228); AKT (1:1000, CST, 4691); p-AKT (1:2000, CST, 4060); GAPDH (1:20000, Proteintech, 60004-1-Ig). Then the membranes were exposed to HRP-conjugated secondary antibody (1:5000, CWbio, CW0102, CW0103) for 1 hour at room temperature. Finally, protein bands were detected using the BeyoECL Plus system (Beyotime, P0018) and band intensities were analyzed with ImageJ software.

**Immunofluorescence staining**

The cells were treated with 5% BSA in PBS for 1 hour before being left overnight at 4°C with the primary antibodies. The next day, the tissue sections or cells on slides were exposed to secondary antibodies for 2 hours and stained with DAPI (Sigma, D9542). Imaging was done using Zeiss laser scanning confocal microscopy and analysis was carried out using ImageJ software. Antibodies used included: mouse anti-GFAP (1:500, CST, 3670), Lamc1 (1:500, Abcam, ab233389), goat anti-rabbit Alexa Fluor 488 (Abcam, ab150077), and goat anti-mouse Alexa Fluor 594 (Abcam, ab150120). After immunofluorescence staining of cultured cells, cell counting was performed using five randomly chosen 10× or 20× visual fields. Cell purity was determined by calculating the ratio of glial cells to total cell count based on DAPI staining.

**Immunohistochemical analysis**

The tissue samples underwent treatment with 3% H_2_O_2_ for 15 minutes prior to being immersed in a blocking solution for 1 hour. After this step, the samples were exposed to a primary antibody (rabbit anti-Lamc1, 1:500, Abcam, ab233389) throughout the night at a temperature of 4°C. Following this, a secondary antibody conjugated to HRP was administered to the samples for 1 hour at a temperature of 37°C. Upon the conclusion of the reaction using 3,3-diaminobenzidine (DAB, Sigma, D8001) and staining with hematoxylin, the samples were visualized utilizing a laser scanning confocal microscope (Zeiss).

**Hematoxylin-eosin (HE) staining**

The samples of spinal cord tissue were first fixed in paraffin and then sliced into 5-μm sections. After being rinsed in distilled water, the sections underwent hematoxylin staining for 5-10 minutes, followed by a wash in tap water for 1-2 minutes and a dip in a solution containing 5% acetic alcohol. The tap water wash continued for more than an hour until the nuclei appeared bright blue, after which there was another rinse in distilled water and staining using a solution consisting of 1% eosin alcohol dye for 3 minutes. To finish, the sections underwent a dehydration process involving incremental alcohol concentrations of 70%, 80%, 95%, and 100%, followed by clearing with xylene and sealing with neutral gum. The sections were then made ready for examination under a microscope to assess the presence of syringomyelia.
